# Nursing management of fatigue in cancer patients and suggestions for clinical practice: a mixed methods study

**DOI:** 10.1186/s12912-021-00699-9

**Published:** 2021-09-28

**Authors:** Angela Tolotti, Loris Bonetti, Carla Pedrazzani, Monica Bianchi, Laura Moser, Nicola Pagnucci, Davide Sari, Dario Valcarenghi

**Affiliations:** 1 Nursing Research and Development Unit, Oncology Institute of Southern Switzerland (IOSI), Via Gallino, 12, 6500 Bellinzona, Switzerland; 2 Department of Business Economics, Health and Social Care, University of Applied Sciences and Arts of Southern Switzerland (SUPSI), Manno, Switzerland; 3 Clinical Trial Unit, Oncology Institute of Southern Switzerland (IOSI), Via Gallino, 12, 6500 Bellinzona, Switzerland; 4 Department of Surgical, Medical and Molecular Pathology and Critical Care, University of Pisa, Pisa, Italy; 5 Department of Nursing, Oncology Institute of Southern Switzerland (IOSI), Via Gallino, 12, 6500 Bellinzona, Switzerland

**Keywords:** Fatigue, Neoplasm, Nurse, Mixed methods

## Abstract

**Background:**

Fatigue is a complex and frequent symptom in cancer patients, influencing their quality of life, but it is still underestimated and undertreated in clinical practice. The aims of this study were to detect the presence of fatigue in cancer patients, describe how patients and nurses perceived it and how nurses managed fatigue.

**Methods:**

This is a mixed methods study. Data were collected in two oncological wards using the Brief Fatigue Inventory (BFI), an ad hoc questionnaire, patient interviews, focus groups with nurses and the review of nursing records. Interviews and focus groups were analysed through thematic analysis. We used SPSS 22.0 for quantitative data and Nvivo 10 for qualitative data analysis.

**Results:**

A total of 71 questionnaires were analysed (39 males, mean age 65.7 years). Fatigue was reported 5 times (7%) in nursing records, while in 17 cases (23.9%) problems associated to it were reported. Twelve patients were interviewed. Five themes were identified: feeling powerless and aggressive, my strategies or what helps me, feeling reassured by the presence of family members, feeling reassured by nurses’ gestures, and being informed. Three themes were identified through the focus groups: objectivity and subjectivity in the assessment of fatigue, nurses’ contribution to the multidisciplinary management of fatigue, and difficulty in evaluating outcomes.

**Conclusions:**

The approach to the management of fatigue was unstructured. Patients were satisfied with the care they received but needed more information and specific interventions. Useful aspects were identified that could be used to change health professionals’ approach towards the management of fatigue.

**Supplementary Information:**

The online version contains supplementary material available at 10.1186/s12912-021-00699-9.

## Background

Fatigue is a very frequent and common symptom in cancer patients, with an incidence ranging between 80 and 100% [1–3]. It is a short- and long-term side effect of most anti-cancer treatments and a consequence of cancer itself, with an onset that may occur at any stage of the cancer disease, from its diagnosis to many years after treatment ends [1, 4–6].

The National Comprehensive Cancer Network (NCCN) [1, 6] defines fatigue as “a distressing, persistent, subjective sense of physical, emotional, and/or cognitive tiredness or exhaustion related to cancer or cancer treatment that is not proportional to recent activity and interferes with usual functioning”.

The aetiology of fatigue is complex and multidimensional and it is rarely an isolated symptom, which makes its management partucularly difficult [1, 4, 6–8].

Fatigue is a symptom that for a long time has been disregarded and underrated, which has had a strong impact on patients’ psychological, physical, and social wellbeing, and therefore on their quality of life, limiting their ability to carry out certain roles, maintain an active social life and cultivate interests and hobbies [9]. Patients describe fatigue as the most destabilizing and distressing symptom [8], which never disappears even with rest and sleep [9]. Therefore, in addition to causing stress and anxiety in patients even for a long time, fatigue may have consequences also for family members or carers [10].

According to the several guidelines on cancer-related fatigue published in the last few years [6], the early screening and assessment of fatigue are key prerequisites for the implemention of individualized and effective patient care [11]. Despite they are strongly recommended in the literature, they are often not routinely performed in clinical practice contributing to their underestimation and undertreatment [1, 3, 5, 12–18]. According to the literature, the main barriers to conducting a structured and complete assessment of fatigue appear to be the lack of precise knowledge about fatigue, its impact on patients, and of the treatment options [16]. In addition, patients and/or their family members often do not report this symptom because they fear that this might entail a reduction or interruption of their treatment, or think fatigue is inevitable and that nothing can be done about it, or simply because they are afraid of complaining too much [19–21].

Therefore, the management of fatigue is a significant challenge for health professionals, and informing and educating patients and their family members to identify and treat it is an integral part of the caring process [1].

In the literature, there is much evidence about the effectiveness of specific interventions [1, 6, 13, 21–23]. There is in particular strong agreement regarding the usefulness of maintaining or promoting moderate physical activity in patients during and after cancer treatment [1, 22]. Due to the multidimensional nature of fatigue, mainly non-pharmacological approaches are recognized to be useful in improving quality of life in these patients during active cancer treatments but also after treatment [1, 24–29]. The importance of an interdisciplinary approach, information and education for patients and relatives is also emphasized [6]. Despite the abundance of literature and guidelines addressing this topic, fatigue is still inadeguately and inconsistently addressed in many institutions [30, 31] where, like in the one where this study was conducted, no standard protocols were in place for the management of fatigue. It is also important to identify effective barriers that lead health professionals to underestimate fatigue and leave it unaddressed in clinical practice, and to understand what are the perceptions of the patients affected by fatigue, to improve its management and the patients’ quality of life. To our knowledge, few studies include an analysis of fatigue based both on the patients’ and the nurses’ perspectives. Furthermore, no mixed method studies were found on this topic.

The aim of the present study was to detect the presence and degree of fatigue in cancer patients, and describe the perceptions of patients and nurses when detecting and assessing this symptom. The secondary objective was to describe how cancer related fatigue is concretely addressed in clinical practice by nurses. In order to have a broader picture and a more in-depth understanding of the phenomenon from different perspectives, we decided to conduct a mixed-method study.

The research questions for this study were:

1. What are the perspectives of patients and nurses regarding the management of fatigue?
2. How can we quantify fatigue in patients and explore to what extent aspects of care and the management of this phenomenon are reported in nursing records?
3. Do the patients’ and nurses’ quantitative and qualitative data regarding their perspectives on the management of fatigue match each other?

## Methods

### Design

This is a mixed-methods study using a convergent parallel design consisting of a patient survey, patient interviews, focus groups with nurses, and the review of nursing records [32, 33]. We adopted a mixed-method design to gain a deeper understanding of fatigue.

### Setting

Two oncology wards of the Oncology Institute of Southern Switzerland (IOSI)*.*

The first ward is an haematological unit with 11 beds and almost 600 admissions per year. The mean length of stay (LOS) is 3 weeks. The nursing team includes 29 health professionals (clinical nurse specialists, registered nurses and nursing aids).

The second ward is a radiotherapy unit, with 13 beds and almost 800 admissions per year. The mean LOS is 2 weeks. The nursing team includes 18 health professionals (clinical nurse specialists, registered nurses and nursing aids).

### Participants

#### Inclusion criteria

- all cancer inpatients able to reply to a questionnaire in Italian and to complete a numerical rating scale;
- nurses working in the two oncology wards of the Oncology Institute of Southern Switzerland (IOSI).

#### Exclusion criteria

- paediatric patients

### Data collection

#### Quantitative data

The questionnaires were distributed and collected by the nurses working in the participating wards. To assess fatigue, we used the Italian version of the Brief Fatigue Inventory (BFI) [34, 35], that showed a good level of validity and reliability (Cronbach’s alpha = .94) [35]. The BFI was administered to patients twice, upon admission and at discharge, to check if the level of fatigue had changed during hospital stay. The research team trained the ward nurses how to collect the data. When an eligible patient was found, the ward nurses illustrated the study, obtained the informed consent, and administered the questionnaire. Patients who were not affected by fatigue according to the BFI upon admission, were excluded from the study. Patients were also asked to complete a questionnaire that investigated how nurses had managed their fatigue, according to the patients’ perception. Through the questionnaire, the patients were asked to state if they had reported their fatigue to the nurses, or if the nurses had detected it. To these two first questions, patients could reply: ‘Never’, “At least once’, or ‘Many times’. Patients were then asked if the nurses had implemented some interventions, which in the literature are reported to be useful to treat fatigue and facilitate rest. To these questions the patients could reply either ‘yes’, ‘partly’, or ‘no’. Finally, they were asked if the nurses had involved other specialists to treat their fatigue. To this question they could reply either ‘yes’ or ‘no’. The questionnaire was pilot tested to check its internal and face validity, and its clarity, with five oncology nurses and five patients, showing a good level of performance with regard to the above characteristics. At the end of the questionnaire, also some of the patients’ demographic data were collected.

To evaluate how fatigue was managed by the nurses, three members of the research team (AT, LB and DV) reviewed the clinical records of the patients included in the study, to identify which focuses were adopted in relation to the patients’ problems. A focus could be a nursing diagnosis, a patient problem, a sign or symptom, a change in the patient’s condition, or any other significant event [36]. Then, we checked which goals and interventions were hypothesized and implemented by the nurses.

#### Qualitative data

Semi-structured interviews and focus groups were conducted to further explore the patients’ and nurses’ experiences about fatigue. The patients were chosen from those who had completed the questionnaires and agreed to be interviewed. The selection was based on the wards, age, and sex, until the number of patients was sufficient to achieve data saturation [32, 37].

The ward nurses identified the elegibile patients and contacted the members of the research team to conduct the interview. The interviews were conducted by two members of the research team (AT and DV)*,* experts in qualitative methods, in the room of the patient, if she/he was alone, or in a quiet dedicated room without external interferences.

The focus groups were conducted with the nurses who were working in the wards involved in the study. The invited nurses were selected by the research team, in agreement with the two head nurses of the unit, who identified which nurses to involve in the study, based on inclusion criteria and availability. Both the patient interviews and focus groups were audio recorded and transcribed verbatim.

### Data analysis

#### Quantitative data

Quantitative data were analysed using SPSS 22.0. A descriptive analysis of the main variables was conducted. The nominal variables were represented in terms of frequency and percentages, and the ordinal or continuous variables respectively in terms of medians, quartiles, means and standard deviations. To compare the groups based on the qualitative variables, the Chi square or Fisher test were used. The Mann Whitney test or t test for unpaired groups were used to compare the ordinal or continuous variables, respectively, if the distribution was normal. We used the Wilcoxon test or the t-test for paired groups to compare the BFI score at the beginning of hospital stay and at discharge. The Kolmogorov-Smirnov normality test was used to determine the normality of the distributions of the continuous variables [37]. The significance level was set at *p* < 0.05.

#### Qualitative data

For the qualitative data, we repeatedly read the whole transcript and then conducted a line-by-line thematic analysis [37–40].

We identified the similarities and differences between the themes related to the patients and those related to the nurses. The analysis was conducted by two expert qualitative researchers, using NVivo 10 software. The methodological rigour of the study was supported by the criteria described by Guba and Lincoln (1981) [41]: credibility, transferability, dependability, confirmability, and fittingness (Carnevale, 2002) [42].

With regard to reflexivity, “bracketing” was adopted to avoid prejudices. The use of a ‘reflective diary’, synthesis, and reflection was helpful to differentiate between what participants said and what researchers understood. Two researchers independently coded the texts of the interviews and the focus groups, to ensure the credibility and transferability of the study. Regarding auditability, all the decisions regarding data collection and analysis made by the researchers were documented using field notes, memos and a reflexivity journal. Fittingness was promoted by various checks with the researchers to ensure that interpretations were consistent with the experiences of the interviewees.

#### Quantitative and qualitative data integration and rigor in relational to mixed methods research

To integrate qualitative and quantitative data, we used a Joint Display. Fetters et al. [43], defined Joint Displays as a way to *“integrate the data by bringing the data together through a visual means to draw out new insights beyond the information gained from the separate quantitative and qualitative results.”*

Following some examples reported in the literature [44–46], we created a table in which we linked qualitative themes with the quantitative results, searching for agreement and disagreement between the two perspectives. Then we added a column with a comment and integrated the findings in a descriptive way.

To guarantee rigor, based on mixed method literature, we used the following legitimation criteria [47, 48]: Commensurability approximation legitimation, Insite-outside legitimation, Integration legitimation, Paradigmatic legitimation, Sample integration legitimation, Sequential legitimation, Socio-political legitimation, Weakness minimisation legitimation. A full description of each of them is reported online in Additional file 1.

## Results

### Survey results

Data were collected from January to November 2017. A total of 71 out of 101 (response rate = 70%) BFI questionnaires were returned upon patient admission, and validly completed to be analyzed. Twenty-two patients completed the questionnaire also at discharge and of these, 20 completed also the questionnaire on the perception of nursing care received in relation to fatigue.

The sample mainly consisted of men (54.9%) and haematological cancer patients (52.1%). The main characteristics of the patients are reported in Table 1.

**Table 1:** Participant characteristics

Characteristics	*N* = 71
**Age- Mean** (± SD)	65.7 (±14)
**Sex (%)**	**n (%)**
M (%)	39 (54.9)
F (%)	32 (45.1)
**BMI** (Me [Q1; Q3])	23.4 [20.8; 27.2]
**LOS** (Me [Q1; Q3])	6 [12; 20]
**Pathology**	**n (%)**
Haematology	37 (52.1)
Solid tumor	33 (46.5)
Both	1 (1.4)
**Fatigue in the physicians’ records**	**n (%)**
Yes	15 (21.1)
No	56 (78.9)
**Fatigue in nurses’ records**	**n (%)**
Yes	5 (7)
No	66 (93)
**Other symptoms related to fatigue in nurses’ records**	**n (%)**
Yes	17 (23.9)
No	54 (76.1)

*Abbreviations*: *BMI* Body Mass Index, *LOS* length of Stay, *Me* median, *Q1* first quartile, *Q3* third quartile, *SD* Standard deviation

A total of 48 participants had experienced episodes of fatigue in the previous 7 days before admission. Fatigue interfered mainly with work activities (median = 5 IQR [2;8]), followed by their ability to walk, physical activity in general, and enjoyment of life. The worst level of fatigue in the last 24 h produced a median score of 7 IQR [5;8]. There were no significant differences in the values of the BFI items for sex, type of cancer or LOS (short = < 5 days; average = 6–15 days; long > 15 days). Neither were there significant differences between the BFI score upon admission and at discharge (Table 2).

**Table 2:** Brief Fatigue Inventory (BFI) results (*N* = 71)

***Tiredness in the last 7 days***^***a***^				**N (%)**							
Yes				48 (66.7)							
No				15 (21.1)							
***BFI scores***				**Mean**		**±SD.**	**Me**	**Q1**			**Q3**
Level of fatigue right now				5.3		2.5	5	3			7
Usual level of fatigue				5.2		2.5	5	3			7
Worst level of fatigue in the last 24 h				6.2		2.5	7	5			8
***How fatigue interfered in the last 24 h with your:***				**Mean**		**±SD.**	**Me**	**Q1**			**Q3**
General activity				4.5		3.3	4	1			8
Mood				3.5		3.1	4	0			6
Walking ability				4.7		3.2	5	2			8
Normal work				4.9		3.4	5	2			8
Relations with other people				3.3		3.1	3	0			6
Enjoyment of life				4.4		3.6	4	1			8
***BFI results admission*vs*discharge (N = 22)***											
	**Admission**					**Discharge**					
***BFI scores***^***a***^	**Mean**	**±SD**	**Me**	**Q1**	**Q3**	**Mean**	**±SD**	**Me**	**Q1**	**Q3**	***p- value***
Level of fatigue right now	4.5	2.7	4.0	2.8	7.0	4.5	2.8	4.0	1.8	7.0	0.8
Usual level of fatigue	4.3	2.6	4.0	2.0	6.3	4.9	2.8	5.5	3.5	7.0	0.3
Worst level of fatigue in the last 24 h	5.2	2.8	5.5	3.0	7.3	5.5	3.1	6.0	4.0	8.0	0.2
***How fatigue interfered in the last 24 h with your:***	**Mean**	**±SD**	**Me**	**Q1**	**Q3**	**Mean**	**±SD**	**Me**	**Q1**	**Q3**	***p- value***
General activity	3.5	3.1	3.0	1.0	5.3	4.3	3.1	5.0	1.0	7.0	0.2
Mood	3.0	3.1	2.0	0.8	4.3	3.1	2.7	2.0	1.0	6.0	0.9
Walking ability	3.7	3.1	3.0	1.0	6.5	3.5	2.9	3.0	0.0	6.0	0.7
Normal work	4.2	3.8	3.5	0.3	8.0	4.1	3.8	3.5	0.0	7.3	0.7
Relations with other people	3.3	3.1	2.5	0.0	6.3	2.9	2.9	2.0	0.0	5.5	0.7
Enjoyment of life	3.6	3.4	2.0	1.0	6.5	2.8	3.1	1.0	0.0	5.8	0.3
**LOS**	10.1	10.0	6.0	3.0	16.5						

*Abbreviations*: *LOS* length of Stay, *Me* median, *Q1* first quartile, *Q3* third quartile, *SD* Standard deviation

^a^missing data

Fatigue was reported 15 times in the medical records. The nurses detected fatigue 5 times, whereas on 17 occasions they reported problems associated with fatigue (e.g. mobility problems, increased soporous state, reduced appetite, and generalized tiredness). In the patient records, no specific interventions implemented by nurses aimed at managing fatigue were found.

With regard to patients’ perceptions of nursing care received, 68.8% reported that nurses had paid attention to their fatigue several times. Regarding actions taken by nurses to deal with fatigue, we found that no information was provided on how to subdivide the day in 45.5% of the cases, reduce efforts in 46.2% of the cases, and measure fatigue autonomously in 54.5% of the cases. Instead, information was provided on how to ask others to do some activities for them in 53.8% of the cases. According to the patients’ perceptions, nurses provided reassurance and psychological support (57.1%), and periodically checked the level of fatigue (46%). In 53.3% of the cases, they did not check or ask what the causes of fatigue could be and in 46.7% of the cases they did not encourage distraction. In 50% of the cases, they implemented interventions for other symptoms, such as pain and dyspnoea. Finally, they tended to avoid requesting consultation services by other professionals (Table 3).

**Table 3:** Questionnaire on patients’ perceptions about care received by nurses to manage their fatigue

**(*N*** **= 20)**^**a**^	**Never** **N(%)**	**At least once N(%)**	**Several times** **N(%)**
**Did you inform the nurses who were caring for you about your fatigue?**	5 (29.3)	4 (23.5)	8 (47.1)
**Do you think the nurses paid attention to your fatigue?**	0	5 (31.2)	11 (68.8)
**Of the actions listed below, were there any that made you feel better and that the nurses performed?**	**Yes** **N(%)**	**Partly** **N(%)**	**No** **N(%)**
**Information/suggestions on how to:**			
➢ Subdivide the day	4 (36.4)	2 (18.2)	5 (45.5)
➢ Reduce efforts	5 (38.5)	2 (15.4)	6 (46.2)
➢ Ask others to do some activities	7 (53.8)	1 (7.7)	5 (38.5)
➢ Measure fatigue by yourself	4 (36.4)	1 (9.1)	6 (54.5)
**Reassurance and/or psychological support**	8 (*57.*1)	2 (14.3)	4 (28.6)
**Periodic check of level of fatigue**	7 (46.7)	2 (13.3)	6 (40)
**Interviews & follow ups to understand the cause of your fatigue**	5 (33.3)	2 (13.3)	8 (53.3)
**Encouraged to distract yourself (e.g.: music, games, reading, etc.)**	5 (33.3)	3 (20)	7 (46.7)
**Help to keep activity levels to the minimum**	5 (33.3)	6 (40)	4 (26.7)
**Involvement of specialists:**	**Yes** **N(%)**	**No** **N(%)**	
➢ For mobilization (physiatrists, physiotherapists)	8 (53.3)	7 (46.7)	
➢ With massages	1 (7.1)	13 (92.9)	
➢ For psychological support (psychologists, etc.)	2 (14.3)	12 (85.7)	
➢ For nutritional support (nutritionists, dietitians, etc.)	4 (26.7)	11 (73.3)	
	**Yes** **N(%)**	**Partly** **N(%)**	**No** **N(%)**
**Interventions to facilitate sleep/rest**	4 (26.7)	5 (33.3)	6 (40)
**Treatment of other symptoms (e.g. pain, dyspnoea …)**	7 (50)	3 (21.4)	4 (28.6)

^a^missing data

### Patients’ perceptions (from interviews)

Between March–November 2017, 12 patients were interviewed, 5 males and 7 females aged between 34 and 82 years.

Five themes were identified: *Theme I: “feeling powerless and aggressive”, Theme II “my strategies or what helps me” Theme III “feeling reassured by the presence of family members”, Theme IV “feeling reassured by nurse’s gestures”, Theme V “being informed”.*

#### Theme I: “Feeling powerless and aggressive”

All the patients names reported in the tables are pseudonyms.

### It changes you, you are different

Patients described fatigue as something that impacted them both physically and psychologically, and influenced their perceptions and senses. They reported how this symptom manifests suddenly, and makes them feel unable to do anything (Table 4, quote 1). Some patients felt powerless, others instead reacted aggressively, although this was unusual for them (Table 4, quote 2). Fatigue manifested at different times of the day, some felt it in the morning, others in the evening, and others throughout the day.

**Table 4:** Quotes Theme I: Feeling powerless and aggressive

*Quote 1, Paolo*^*a*^*: “it started suddenly without a reason, I no longer do anything, almost as if I went in hibernation. I’m just the ghost of myself.”* *Quote 2, Simona: “I had an episode of aggressiveness, by nature I’m not an aggressive person, but for a silly thing I suddenly flared up and shouted!”.”* *Quote 3, Giulia: “you no longer feel like reading, like taking your life in your hands, you no longer feel like going to the supermarket, you choose a time when there’s no-one, because when you are unwell everything seems too much”* *Quote 4, Paola “there were times when I couldn’t go out of my home. It was tremendous because I’ve always been independent.”* *Quote 5, Elena: “I’m a teacher and I tried to mark my students’ work, but I realized that when I edited the file, I no longer remembered what my student had written.”*	

^a^All the patients’ names reported in the tables are pseudonyms

### Influences habits and social relations

Patients reported how strenuous it was for them to carry out their daily living activities or to stay in places where there were many people, such as supermarkets. Everything became hard and undesirable, described as ‘too much’ for their physical and emotional resources (Table 4, quote 3).

Fatigue and the disease also significantly changed the person’s roles in terms of relations, both in daily life and at work. People ended up depending on others and this caused psychological suffering (Table 4, quote 4).

Some patients also reported about problems with concentrating and short-term memory, which impacted also on their jobs (Table 4, quote 5).

#### Theme II “My strategies or what helps me”

Some patients thought that forcing themselves to do certain things, like going up the stairs, doing yoga, some manual work, and going out for a walk, helped them to cope with tiredness. They reported that it was also important to ‘throw it all out’, like shouting for instance.

The need to rest was considered to be important, as well as maintaining the normal sleep-wake cycle (Table 5, quote 1).

**Table 5:** Quotes for Theme II: “My strategies or what helps me”

*Quote 1, Simona “when I’m tired, I go up and down the stairs a couple of times, I try to make the effort, then I rest, I do yoga breathing, I shout out, and throw it all out”* *Quote 2 Lory “It is important to act on will power, I try to stand up from the chair and if fatigue arrives, I sit down and wait until it passes away or gets better. In these moments, I try to read or listen to music, and think that it will go away just like it came.”* *Quote 3, Giulia “I listen to my body very much, I try to avoid what is not good, whatever weighs down on me or negative people. I do some cleaning.”*	

Some reported that the only thing they could do was to act on will power and do some physical exercise, like standing up from the chair. When fatigue arrives, some patients said: “I sit and wait”, as if all they could do was wait until it passed away or got better (Table 5, quote 2).

When fatigue prevails, you end up having bad thoughts, therefore it is important to listen to your own body, avoiding negative things or people, and concentrating on positive things (Table 5, quote 3).

#### Theme III “Feeling reassured by the presence of family members”

Family members and friends are a very important resource (Table 6, quote 1).

**Table 6:** Quotes for Theme III: “Feeling reassured by the presence of family members”

*Quote 1, Paolo “I have a fantastic wife, she is very active and also the kids, they are there whenever I’m in need, they are an important reference point. At home my wife lets me do things my way. She understands me.”* *Quote 2 Agata: “I have a wonderful family … my husband … my daughters … my best friend, I call her my coach. My nephews and nieces are tremendous … their smiles give me energy.*	

Receiving visits from friends or relatives is important because ‘you talk about other things’, as if they felt the need to shift their attention from something that caused pain and immobility to ‘other things’. Patients reported that when they feel ‘melancholic’ they feel comforted and strong thanks to the presence and support of their family members and friends. Being surrounded and at the centre of other’s affection and attention was very important for the patients (Table 6, quote 2).

#### Theme IV: “Feeling reassured by nurses’ gestures”

##### Nurses’ gestures

Nurses made patients feel reassured, the caring expression in their eyes showed an authentic interest and were able to capture your needs even when these were not evident (Table 7, quote 1). Patients underlined that the nurses’ interest was perceived to be authentic when it was accompanied by concrete gestures (Table 7, quote 2).

**Table 7:** Quotes for Theme IV: “Feeling reassured by the nurses’ gestures”

*Quote 1, Anna “they worry, look, check, ask what you. They are always there, more than the doctors, and they reassure you”* *Quote 2 Gionata “they come back to see how you are, they try to find solutions, they take me for a walk”.* *Quote 3 Ginevra “As a patient you understand who is the right person and with whom you can open up with. Ok that person speaks like me … you notice it from the person’s attitude”* Quote 4 Giovanni *“the focus is not just on the person’s feelings, but also on the life that is out there”.* Quote 5 Simona *“they don’t have much time because they’re busy doing other things and there are people who are worse off than us. I think it’s not easy to manage all this, but I think that in the future they will need to think also of those who feel a bit better”.* Quote 6 Agata*“Nurses have many things to do and I think: “fatigue is difficult to deal with, for them it is an extra thing”.*	

Talking to nurses about one’s fears and feelings was considered to be important, but patients also underlined that before opening yourself up to nurses, you must be able to trust them and make sure that you are understood (Table 7, quote 3). Also talking about things that ‘do not concern the hospital’, like sports events was important (Table 7, quote 4).

Patients wanted to receive advice from nurses on how they could manage fatigue but this did not always happen, and according to the patients this was because nurses did not have enough time or had to give the priority to patients who were ‘feeling worse than them” (Table 7, quote 5).

With regard to fatigue, some patients reported that nurses considered the onset of this symptom ‘normal’ or a ‘routine’; others considered it something odd they had to deal with (Table 7, quote 6).

When nurses collected data on fatigue, patients felt they were being ‘taken in charge’. Some patients underlined the importance of this study and wished that more attention would to be placed on this symptom.

Some patients reported that they had been encouraged to walk, and considered physicians and nurses as their “sports coach”. This portrays the competence of health professionals who know how to help these patients achieve important goals, by working on a person’s ability, motivation and will power.

Other patients reported that although they did speak to nurses, they did not receive any advice on how to manage fatigue. They remember that sometimes nurses remained in silence, an attitude that was interpreted as ‘they are reflecting, they are thinking about it’. Some patients justified the nurses for not giving them any advice, thinking that fatigue ‘is not a problem that is easy to resolve’.

Others thought that specialized nurses could go more in depth and provide practical advice on how to manage their psychophysical resources.

#### Theme V “Being informed”

Being informed about fatigue, knowing its causes and how to control it was very important for patients and helped them play an active role in the care process and understand in advance when certain events may occur (Table 8, quote 1).

**Table 8:** Quotes for Theme V: “Being informed”

*Quote 1, Ginevra “being informed enables me to have a more commensurate role with nurses, information /knowledge helps me to anticipate things before they happen”.* *Quote 2, Anna “Receiving accurate and timely answers is very important, being informed made me feel involved”.* *Quote 3 Claudia “I asked many things both to physicians and nurses, and I have to say that they were very helpful and answered to my questions. However, I felt the need to gain a deeper understanding, I searched the Internet a lot and then I did things my own way, with all the limitations this involved”* *Quote 4 Simona, “The information I would need has to more accurate, structured and monitored. In this I felt a little abandoned”.*	

Some patients were satisfied with the information they had received and considered the exchange of information between physicians and nurses a positive element (Table 8, quote 2).

Other patients thought that the information they had received was not complete, checked, and not enough time dedicated to providing it, giving rise to a sensation of abandonment (Table 8, quote 3). When information was not deemed sufficient, patients consulted other sources (Table 8, quote 4).

### The perception of nurses (from the focus groups)

A total of 15 nurses, (3 men and 12 women, aged between 25 and 45) years old took part in two focus groups.

We identified three major themes and their subthemes: *Theme I: ‘Objectivity and subjectivity in the assessment of fatigue’, Theme 2: ‘Nursing contribution in the multidisciplinary management of fatigue’ and Theme 3: ‘Difficulty evaluating outcomes’.*

#### Theme I: Objectivity and subjectivity in the assessment of fatigue

The term used by nurses to describe fatigue was ‘tiredness’, physical and mental, in some cases makes patients feel unmotivated to do things (Table 9, quote 1).

**Table 9:** Themes and quotes from the two focus groups with the nurses

Theme	Quotes
*Theme I:* *“Objectivity and subjectivity in the assessment of fatigue”*	1. *“It’s a kind of tiredness that leaves patients with very little willingness to face the day, with little motivation” (Focus Group 1, Nurse 1)* 2. *“Sometimes, during handover you receive information about how the nurse who was there before you perceived the patient, but then you personally think that the patient is no longer like that. Maybe because the patient is feeling better now or we may have different ways of seeing things” (Focus Group 2, Nurse 4)* *“Ours is a perception” (Focus Group 1, Nurse 3)*
*Theme II:* *“Nursing contribution in the multidisciplinary management of fatigue”*	*3. “We have to reflect on this because I think that in addition to encouraging patients to do things, we are mostly their spokespersons” (Focus Group 2, Nurse 5)* *4. “Sometimes relatives insist a lot and we let them do things we are not supposed to let them do” (Focus Group 2, Nurse 3)* *“I saw the case of a patient where his wife was present and helped out” (Focus Group 1, Nurse 4)* *5. “When we listen to a patient that talks about this symptom it’s a problem for us, sometimes we really don’t know what to do” (Focus Group 2, Nurse 6)* *6. “We do the interventions but in my view, they are just our own initiative, and we don’t always feel like talking about them, or writing a report about them or objectifying them” (Focus Group 2, Nurse 1)* *“the thing it that we don’t agree on a care plan with our colleagues, we just say: there’s this problem, what shall we do? Each of us does a little of what has already been done before or seen before. There is no thinking about which interventions need to be implemented in the future.” (Focus Group 1, Nurse 8)*
*Theme III:* *“Difficulty evaluating outcomes”*	*7.“Frankly, I’m not sure of the actual benefit I am providing, it may well be an inducted symptom that it could be due to the patient’s treatment”. (Focus Group 1, Nurse 5)* *“We experience a bit of frustration, in the sense that we do what we can, but what he actually does is that he just stays in bed”. (Focus Group 2, Nurse 7)*

Nurses underlined how this problem was very frequent due to the ‘type of patients’, who were not ‘fit’ due to their age and the ‘intensive’ treatments. Assessing the presence of fatigue was described by nurses as a ‘subjective perception’, and this generated uncertainty (Table 9, quote 2).

The aspects taken into account were how patients carried out their activities of daily living, how they greeted you, and their willingness to talk. The possibility of considering measurable ‘objective’ data (e.g. blood tests, vitals signs, etc.) was also considered important. The collection of these data would lead nurses to include the ‘fatigue focus’ [36] in the nursing records, where a daily assessment would be made to monitor the course of this symptom. In relation to this, some of the nurses reported that they did not use the ‘fatigue focus’ but rather a focus on what they define as ‘something similar to it’ (e.g. difficulty moving, or lack of appetite). At times, patients declared that they were feeling weak, describing it as ‘a feeling of uneasiness’. The nurses underlined that fatigue is something that they discuss about with other nurses, but rarely during multidisciplinary meetings.

#### Theme II: “Nursing contribution in the multidisciplinary management of fatigue”

This theme included two subcategories: those who deal with fatigue, and the nursing management of fatigue.

##### Those who deal with fatigue

The nurses reported that it was important to ensure the multidisciplinary management of fatigue. According to the nurses, the most appropriate health professionals for this are physiotherapists and dieticians. The nurses also declared that nursing plays a key role in the multidisciplinary management of fatigue, but they had two different views about it: some reported that nurses had to be proactive; others instead saw themselves as playing the role of the patient’s ‘spokesperson’. The nurses debated on this aspect and agreed that it is important to reflect on what is currently done and on what instead could be done (Table 9, quote 3).

The patient’s family was also described as an important resource, although nurses had divergent feelings about this, because on one hand families helped, but on the other, their presence can at times be ‘cumbersome’ (Table 9, quote 4).

##### The nursing management of fatigue

Fatigue was described as a multifactor symptom, which nurses considered complicated to manage (Table 9, quote 5).

Nurses described interventions such as encouraging patients to take care of their personal hygiene and to get out of bed. They reported that it was important to allow *“all the time they needed”* but also to *“encourage them”.* In addition, also a better organization of the space available in the patient’s room could facilitate the patient’s movements.

However, nurses reported that these types of interventions were implemented on their own initiative (Table 9, quote 6). Therefore, there were various ways of addressing fatigue, some nurses focused more on encouraging patient mobility and others on nutrition, almost as if the interventions depended on the nurses’ individual sensations or a personal way of viewing the problem.

Other than the interventions made by the nurses, there was also an ‘organizational’ aspect regarding the procedure to request the specific intervention of other health professionals, such as physiotherapists, dieticians or psychologists.

#### Theme III: “Difficulty evaluating outcomes”

The process adopted by the nurses to evaluate the outcomes was poorly structured. The aspects described by the nurses were essentially two: when the patients report that they no longer feel cold and start joking and smiling.

The nurses also underlined the difficulty in evaluating if an outcome was actually the consequence of a nursing intervention or the result of radiotherapy or chemotherapy for instance, and this sometimes led to a sense of frustration (Table 9, quote 7). To address these difficulties, the nurses claimed that it would be useful for them to act upon three aspects: education, use validated tools to measure fatigue, and know the results of the present study.

Finally, the nurses suggested that also the availability of a set of guidelines for an objective assessment of fatigue would be very useful to evaluate the outcomes of their interventions.

### Integration of the qualitative and quantitative findings

The Joint display that integrate the qualitative and quantitative findings is reported in Table 10.

**Table 10:** Joint display for qualitative and quantitative data integration

**QUANTITATIVE RESULTS**	**QUALITATIVE RESULTS (PATIENTS INTERVIEWS)**	**QUALITATIVE RESULTS (NURSES FOCUS GROUPS)**	**AGREEMENT POINTS**	**DISAGREEMENT POINTS**	**CONCLUSIONS**
**BFI questionnaire**	**THEMES**	**THEMES**	**X**		Both quantitative and qualitative data show that it is an important issue for patients and nurses, which has a strong impact on quality of life.
Fatigue and work activities median = 5	FEELING POWERLESS AND AGGRESSIVE It changes.- you are different- influence habit and social relations	OBJETIVITIES AND SUBJECTIVITIES IN THE ASSESSEMENT OF FATIGUE			
**Patient perception nurses’ care received:**	**QUALITATIVE RESULTS (PATIENTS INTERVIEWS)**	**QUALITATIVE RESULTS (NURSES FOCUS GROUPS)**	**AGREEMENT POINTS**	**DISAGREEMENT POINTS**	**CONCLUSIONS**
68.8% of the nurses paid attention to patient’s fatigue	FEELING REASURED BY THE NURSES’ GESTURES BEING INFORMED	NURSING CONTRIBUTION IN THE MULTIDISCIPLINARY MANAGEMENT OF FATIGUE	**X**		Qualitative and quantitative data confirm that nurses pay attention to the fatigue syndrome in patients, despite nurses do not always report the actions they implement to manage fatigue and are scarcely documented.
53.8% Nurses provided information on how to ask others to do some activities for them	FEELING REASURED BY THE PRESENCE OF FAMILY MEMBERS BEING INFORMED	Those who deal with fatigue	**X**		Both the quantitative and the qualitative data (of patients and nurses) underline the importance of caregivers.
57.1% of patients perceived to have received psychological support	FEELING REASURED BY THE NURSES’ GESTURES		**X patient quantitative and qualitative data**		Regarding this aspect, there is agreement between patients’ quantitative and qualitative data. Nurses do not mention about psychological support for the management of the disease. Probably because this type of psychological support was provided by health professions other than nurses, or because they gave it for granted.
53.8% no information provided by nurses on how to measure fatigue autonomously	BEING INFORMED		**X**		There is agreement between quantitative and qualitative data because the patients confirmed, also in the qualitative data, the importance of being informed. However, this information was sometimes incomplete or was provided only to some patients.
45.5% no information provided by nurses on how to subdivide the day	BEING INFORMED				
46.2% no information provided by nurses on how to reduce efforts	BEING INFORMED				
46% of the time nurses checked patients for fatigue	FEELING REASURED BY THE NURSES’ GESTURES	OBJECTIVITY AND SUBJECTIVITY IN THE ASSESSMENT OF FATIGUE		**X**	There is inconsistency between what patients and what nurses perceived in relation to the assessment of fatigue. Patients perceived that they had been assessed for fatigue few times, whereas nurses said that they do this assessment, although they underline the difficulty of conducting an objective assessment.
**Patients’ records** Difference in reporting fatigue by physicians (15 times) and nurses (5 times)		NURSING CONTRIBUTION IN THE MULTIDISCIPLINARY MANAGEMENT OF FATIGUE The nursing management of fatigue DIFFICULTY EVALUATING OUTCOMES		**X**	Despite nurses pay attention to fatigue (see positive aspects), in reality it is documented very little in the patients’ records and it is sometimes described through other symptoms (nutrition, movement/ exercise, etc.)

## Discussion

The aim of this study was to detect the presence and the intensity of fatigue in hospitalised cancer patients and to describe the perceptions of patients and nurses when they detect and assess this symptom.

In general, there was agreement between patients’ and nurses’ perspectives, and between quantitative and qualitative data. The only two points for which there was disagreement were: a) the assessment of fatigue, where patients perceived to have received less fatigue assessment compared to what nurses thought they had performed; and b) the management of fatigue, where there was a discrepancy between what nurses said and what was actually reported in the patients’ records (Table 10, Joint display). These discrepancy is consistent with the literature, where fatigue is reported to be still inadeguately and inconsistently addressed by health professionals in many institutions [30, 31], underlining the importance of adopting a more standardized approach.

The results of the present study showed that patients had already developed fatigue also during the seven days prior to its detection (66%). Both patients and nurses were aware that fatigue had a physical and psychological impact, thus compromising their ability to keep up with the normal activities of everyday life. Through the questionnaire, the patients described how fatigue interfered with their work activities and with the activities of daily life in general. This finding was also reported by Wu & Davis [49] and Borneman et al. [9].

Participants described how the onset of fatigue was ‘sudden’ and unrelated to a precise cause, and was often a source of frustration and anger, as also reported by Scott et al. [19]. The nurses reported that detecting the onset of fatigue was important, but at the same time very difficult to perform and assess in an ‘objective’ manner. This difficulty also emerged from the analysis of the nursing documentation regarding the patients included in this study. In fact, there were inconsistencies in the way fatigue was assessed across medical records (15 times) and across nursing records (5 times), as already stated. In the nursing records, we noticed that fatigue was not always reported, but rather the problems that were related to it, such as physical exercise, rest and sleep, or loss of appetite (17 times). As reported in the literature [1, 6, 30, 31] we also found that nurses tended to underestimate the perception of fatigue more than patients. One possible reason for this could be the widespread belief that it is an unavoidable, complex symptom for which you do not really know what to do. (Table 9, quote 5).

The strategies adopted by the patients to deal with fatigue, such as physical activity or changing their sleep/wake rhythms, were similar to those described by Borneman [9] and recommended in the literature, but the innovative element reported by some of our patients was the importance of acting on will power, concentrating on positive things and removing negative thoughts or situations.

For the patients, it was very important to be informed about fatigue and how to deal with it, because this helps them to find the right strategies and play an active role in managing this symptom. However, the information provided by nurses and physicians was not always considered to be sufficient and as a consequence they sometimes consulted other sources (e.g. the Internet) as also reported in other studies [50–52]. Information was important also for nurses, who underlined a twofold difficulty. On one hand, the difficulty identifying effective interventions on which everyone agreed for the management of this symptom; and on the other, the difficulty in playing a proactive role within the multidisciplinary team to attract more attention on the need to consistently assess and manage fatigue.

The presence of nurses was very important for our patients because they made them feel reassured, and free to talk openly of what they experienced and their fears, similarly to another study [52]. Patients wanted to receive more advice from nurses, but this did not always happen, and patients thought that this was due to the nurses’ lack of time and the actual difficulty in dealing with fatigue. As mentioned above, also nurses thought it was difficult to intervene effectively on fatigue and declared that those interventions they do implement were often the result of subjective initiatives rather than a structured planned intervention. In addition, nurses also declared that it was difficult to evaluate the outcomes of the interventions that addressed fatigue due to the lack of validated tools.

Mackereth et al. [50] describe the patients’ frustration for their family members’ inability to understand their fatigue, instead this aspect did not emerge from our study. In fact, our patients said that it was very important to be with their family members and friends, because they felt understood and supported by them. The nurses also thought that the family’s support was very important for these patients and underlined their willingness to listen to their requests and observations, and involve them in the care process. Therefore, the involvement of family members (and/or friends) in the management of this symptom over time appears to be a relevant aspect.

### Limitations

This study has some limitations that need to be considered. Firstly, this was a monocentric study linked to a specific context, therefore the results should be interpreted in the light of this. Furthermore, we did not calculate the sample size for the quantitative data. Secondly, the patients’ records were very difficult to consult because data were fragmented and not easy to find, therefore some relevant data may have been missed. Moreover, it is likely that the nurses did not report all the measures they adopted to manage fatigue and the paucity of reported interventions could be the consequence of this. Finally, only 22 patients responded to the BFI questionnaire at discharge, and only 20 patients responded to the questionnaire about perceived nursing care received to deal with their fatigue, and this may have limited our results.

### Implications for clinical practice and nursing

Despite fatigue is a widespread symptom and its impact on cancer patients is significant, it still tends to be underestimated by health professionals. As suggested by the participants of this study, to bridge this gap it is necessary to raise major awareness in health professionals and increase their knowledge about fatigue, as well as their ways of treating it. With regard to health professionals’ awareness, it could be helpful to reflect together with them on the results of the present study (as well as other studies), thus simulating a reflective thinking approach. Instead, with regard to health professionals’ knowledge, it would be appropriate to conduct specific interprofessional educational sessions on fatigue, and to take into account the patients’ indications (i.e. having appropriate advice and being supported in their motivation).

With regard to nurses’ practice, the various phases of the nursing process need to be improved, starting from the assessment, to the interventions, multi-professional collaboration and outcome evaluation. Once all these aspects have been clarified and negotiated, they could constitute a specific guideline to be implemented within the health organization and then periodically updated.

## Conclusions

When comparing the perceptions of the patients with those of the nurses who were caring for them, we identified some interesting aspects that could be used to change health professionals’ approach to the way they manage fatigue, in addition to what the literature recommends.

The patients adopted various personal strategies that helped them cope with fatigue, but they also suggested to the nurses some possible interventions that could be helpful for them. On the other hand, nurses could gain important knowledge from scientific evidence and by listening to their patients and improve the effectiveness of their professional practice. Reviewing and discussing these data together with the team of health professionals that produced them, could help them become more aware of the issues related to fatigue and facilitate the change process within their organization.

## Supplementary Information

**Additional file 1.** Legitimation criteria.

## Data Availability

The data and all supporting materials used in our manuscript are freely available to any scientist wishing to use them from the corresponding author on request.

## Abbreviations

BFI: Brief fatigue inventory
SPSS: Statistical package for the social sciences
NCCN: National Comprehensive Cancer Network
Q1: First quartile
Q3: Third quartile
IQR: Iterquartile range
SD: Standard deviation
BMI: Body mass index
LOS: Length of stay
Me: Median
