# Supplementary material for: Nursing management of fatigue in cancer patients and suggestions for clinical practice: a mixed methods study
Source: BMC Nurs. 2021 Sep 28;20:182. doi: 10.1186/s12912-021-00699-9 (PMC8477483; doi:10.1186/s12912-021-00699-9)
Supplement: Supplementary file 1 — Additional file 1. Legitimation criteria. [file 12912_2021_699_MOESM1_ESM.docx]

| **Additional file 1. Legitimation criteria** | |
| --- | --- |
| **Legitimation type** | **Strategies used** |
| Commensurability approximation legitimation | The research was conducted by experienced researchers in qualitative, quantitative, and mixed methods.  The qualitative approach was used to investigate the emic perceptive and the quantitative methodology focused on etic views. The expert in mixed methods assured a balanced account of both views. |
| Inside-outside legitimation | Quantitative data and quotes from qualitative phase were summarized in the Joint Display.  To explore the emic perspective (patients’ and nurses’ views) we used inductive thematic analysis.  To ensure inter-rater reliability two researchers independently coded the texts of the interviews and the focus groups.  To maintain a balance between emic and etic views the researchers analyzed qualitative and quantitative data together. |
| Integration legitimation | In all the study phases, the authors tried to integrate the quantitative and the qualitative data. This is shown in the Joint Display. |
| Paradigmatic legitimation | The research questions guided the researchers in choosing the most suitable methodologies to decide the phases of the study, and the data collection methods and analysis.  We asked ourselves the following questions:   - Should we use a validated questionnaire to assess fatigue? - Should we use an inductive or deductive analytic approach?   The research team agreed to use a validated questionnaire present in the literature and an inductive thematic analysis was adopted to understand the perceptions of patients and nurses. |
| Sample integration legitimation | With regard to the quantitative data, we used a convenience sample, which was not representative. Our intent was not to achieve generalizability but to evaluate these variables in our context.  For the qualitative phase, the sample was convenient and purposive, but variability was reasonable. |
| Sequential legitimation | The research team agreed to use a convergent parallel design because a validated questionnaire was already available for the assessment of fatigue, whereas patients’ and nurses’ perceptions about this phenomenon have been scarcely investigated. |
| Socio-political legitimation | The results of our study highlighted the importance of improving the assessment and treatment of fatigue through the perceptions of patients and nurses. By reflecting on the results of our study, there could be the opportunity to improve nursing interventions to assess and manage patient fatigue.  This study offers useful information for both stakeholders. In addition, knowledge about the patients’ perspective of the problem could help nurses find more effective and personalized strategies to address the problem. |
| Weakness minimisation legitimation | For the quantitative data we used a validated tool (BFI), for the ad hoc questionnaire we conducted a pilot test to assess its reliability and validity. For the qualitative data we used Guba and Lincoln’s criteria, and for data integration we used the joint display.  Through these methods we tried to minimize the limits of each approach, trying to achieve the added value of gaining further insight into the problem, which would have been difficult to achieve if two separate approaches had been used. |

(Legitimation criteria, adapted from: Onwuegbuzie & Johnson (2006) The validity issue in mixed research. Research in the Schools, 13(1), 48-63 & Younas A, Parveen Rasheed S, Zeb H. Using legitimation criteria to establish rigour in sequential mixed-methods research. Nurse Res. 2020 Sep;28(3):44–51).
